# Black and south asian women’s pathways to accessing community and inpatient perinatal mental health services: An analysis of local service data from the paam study

**DOI:** 10.1192/j.eurpsy.2021.1611

**Published:** 2021-08-13

**Authors:** N. Jovanovic

**Affiliations:** Unit For Social And Community Psychiatry, Queen Mary University of London, London, United Kingdom

**Keywords:** Ethnicity, perinatal, Access, Pathways

## Abstract

**Introduction:**

Women from ethnic minorities who experience mental health problems during the perinatal period are disproportionately represented in involuntary care. They have poorer access to community care but have higher engagement with services once accessed. Their pathways to accessing perinatal mental health care remain underexplored.

**Objectives:**

To investigate the pathways to perinatal mental health services for women across different ethnic groups, including number of caregivers encountered and time elapsed between referrals.

**Methods:**

Analysis of patient records and routine service data from community and inpatient perinatal mental health services in the United Kingdom. Use of an adaptation of the WHO’s pathway encounter form.

**Results:**

Women from ethnic minority groups experience increased levels of complexity on their journey to accessing perinatal mental health care. We will present a detailed analysis of patient and service characteristics.

**Conclusions:**

Referral pathways to perinatal mental health services need to be optimised for women from underrepresented groups.

